# Supplementary material for: Effect of Pharmacoprophylaxis on Postoperative Outcomes in Adult Elective Colorectal Surgery: A Multi-Center Retrospective Cohort Study within an Enhanced Recovery after Surgery Framework
Source: Healthcare (Basel). 2023 Nov 28;11(23):3060. doi: 10.3390/healthcare11233060 (PMC10706554; doi:10.3390/healthcare11233060)
Supplement: Supplementary file 1 [file healthcare-11-03060-s001.zip › healthcare-2701432-supplementary.pdf]

Supplemental Materials Figure S1 – CPT and ICD-10 codes connection to colorectal surgery

Common procedural codes (CPT) for colorectal surgeries

Colectomy

Open procedures (44140-44160)
44140 Colectomy, partial; with anastomosis
44141 Colectomy, partial; with skin level cecostomy or colostomy
44143 Colectomy, partial; with end colostomy and closure of distal segment (Hartmann type procedure)
44144 Colectomy, partial; with resection, with colostomy or ileostomy and creation of mucofistula
44145 Colectomy, partial; with coloproctostomy (low pelvic anastomosis)
44146 Colectomy, partial; with coloproctostomy (low pelvic anastomosis), with colostomy
44147 Colectomy, partial; abdominal and transanal approach
44150 Colectomy, total, abdominal, without proctectomy; with ileostomy or ileoproctostomy
44151 Colectomy, total, abdominal, without proctectomy; with continent ileostomy
44155 Colectomy, total, abdominal, with proctectomy; with ileostomy
44156 Colectomy, total, abdominal, with proctectomy; with continent ileostom
44157 Colectomy, total, abdominal, with proctectomy; with ileoanal anastomosis, includes loop ileostomy, and rectal mucosectomy, when performed
44160 Colectomy, partial, with removal of terminal ileum with ileocolostomy

Laproscopic procedures (44204-44346)
44188 Laparoscopy, surgical, colostomy or skin level cecostomy
44204 Laparoscopy, surgical; colectomy, partial, with anastomosis
44205 Laparoscopy, surgical; colectomy, partial, with removal of terminal ileum with ileocolostomy
44206 Laparoscopy, surgical; colectomy, partial, with end colostomy and closure of distal segment (Hartmann type procedure)
44207 Laparoscopy, surgical; colectomy, partial, with anastomosis, with coloproctostomy (low pelvic anastomosis)
44208 Laparoscopy, surgical; colectomy, partial, with anastomosis, with coloproctostomy (low pelvic anastomosis) with colostomy
44210 Laparoscopy, surgical; colectomy, total, abdominal, without proctectomy, with ileostomy or ileoproctostomy
44211 Laparoscopy, surgical; colectomy, total, abdominal, with proctectomy, with ileoanal anastomosis, creation of ileal reservoir (S or J), with loop ileostomy, includes rectal mucosectomy, when performed
44212 Laparoscopy, surgical; colectomy, total, abdominal, with proctectomy, with ileostomy
44213 Laparoscopy, surgical, mobilization (take-down) of splenic flexure performed in conjunction with partial colectomy (to be listed separately in addition to primary procedure
44320 Colostomy or skin level cecostomy
44346 Revision of colostomy; with repair of paracolostomy hernia (separate procedure)

Rectal procedures (45110-45550)

45110 Proctectomy; complete, combined abdominoperineal, with colostomy
45111 Proctectomy; partial resection of rectum, transabdominal approach
45112 Proctectomy, combined abdominoperineal, pullthrough procedure (eg, colo-anal anastomosis) 45113 Proctectomy, partial, with rectal mucosectomy, ileoanal anastomosis, creation of ileal reservoir (S or J), with or without loop ileostomy
45114 Proctectomy, partial, with anastomosis; abdominal and transsacral approach
45116 Proctectomy, partial, with anastomosis; transsacral approach only (Kraske type)
45119 Proctectomy, combined abdominoperineal pullthrough procedure (eg, colo-anal anastomosis), with creation of colonic reservoir (eg, J-pouch), with diverting enterostomy when performed
45120 Proctectomy, complete (for congenital megacolon), abdominal and perineal approach; with pull-through procedure and anastomosis (eg, Swenson, Duhamel, or Soave type operation)

45121 Proctectomy, complete (for congenital megacolon), abdominal and perineal approach; with subtotal or total colectomy, with multiple biopsies
45123 Proctectomy, partial, without anastomosis, perineal approach
45126 Pelvic exenteration for colorectal malignancy, with proctectomy (with or without colostomy), with removal of bladder and ureteral transplantations, and/ or hysterectomy, or cervicectomy, with or without removal of tube(s), with or without removal of ovary(s), or any combination thereof
45130 Excision of rectal procidentia, with anastomosis; perineal approach
45135 Excision of rectal procidentia, with anastomosis; abdominal and perineal approach
45136 Excision of ileoanal reservoir with ileostomy
45150 Division of stricture of rectum
45160 Excision of rectal tumor by proctotomy, transsacralortranscoccygeal approach
45171 Excision of rectal tumor, transanal approach; not including muscularis propria (ie, partial thickness)
45172 Excision of rectal tumor, transanal approach; including muscularis propria (ie, full thickness)
45190 Destruction of rectal tumor (eg, electrodesiccation, electrosurgery, laser ablation, laser resection, cryosurgery) transanal approach
45395 Laparoscopy, surgical; proctectomy, complete, combined abdominoperineal, with colostomy
45397 Laparoscopy, surgical; proctectomy, combined abdominoperineal pull-through procedure (eg, colo-anal anastomosis), with creation of colonic reservoir (eg, J-pouch), with diverting enterostomy, when performed
45400 Laparoscopy, surgical; proctopexy (for prolapse)
45402 Laparoscopy, surgical; proctopexy (for prolapse), with sigmoid resection
45540 Proctopexy (eg, for prolapse); abdominal approach
45541 Proctopexy (eg, for prolapse); perineal approach
45550 Proctopexy (eg, for prolapse); with sigmoid resection, abdominal approach

Robotic-assisted procedures

S2900 Surgical techniques requiring use of robotic surgical system (list separately in addition to code for primary procedure)

ICD-10-PCS PROCEDURE CODE PROCEDURE CODE DESCRIPTION

PARTIAL EXCISION OF COLON

0DBE0ZZ	Excision of large intestine, open approach
0DBF0ZZ	Excision of right large intestine, open approach
0DBG0ZZ	Excision of left large intestine, open approach
0DBH0ZZ	Excision of cecum, open approach
0DBK0ZZ	Excision of ascending colon, open approach
0DBL0ZZ	Excision of transverse colon, open approach
0DBM0ZZ	Excision of descending colon, open approach
0DBN0ZZ	Excision of sigmoid colon, open approach
0DBE4ZZ	Excision of large intestine, percutaneous endoscopic approach
0DBF4ZZ	Excision of right large intestine, percutaneous endoscopic approach
0DBG4ZZ	Excision of left large intestine, percutaneous endoscopic approach
0DBH4ZZ	Excision of cecum, percutaneous endoscopic approach
0DBK4ZZ	Excision of ascending colon, percutaneous endoscopic approach
0DBL4ZZ	Excision of transverse colon, percutaneous endoscopic approach
0DBM4ZZ	Excision of descending colon, percutaneous endoscopic approach
0DBN4ZZ	Excision of sigmoid colon, percutaneous endoscopic approach

TOTAL EXCISION OF COLON

0DTE0ZZ	Resection of large intestine, open approach

0DTF0ZZ	Resection of right large intestine, open approach
0DTG0ZZ	Resection of left large intestine, open approach
0DTH0ZZ	Resection of cecum, open approach
0DTK0ZZ	Resection of ascending colon, open approach
0DTL0ZZ	Resection of transverse colon, open approach
0DTM0ZZ	Resection of descending colon, open approach
0DTN0ZZ	Resection of sigmoid colon, open approach
0DTE4ZZ	Resection of large intestine, percutaneous endoscopic approach
0DTF4ZZ	Resection of right large intestine, percutaneous endoscopic approach
0DTG4ZZ	Resection of left large intestine, percutaneous endoscopic approach
0DTH4ZZ	Resection of cecum, percutaneous endoscopic approach
0DTK4ZZ	Resection of ascending colon, percutaneous endoscopic approach
0DTL4ZZ	Resection of transverse colon, percutaneous endoscopic approach
0DTM4ZZ	Resection of descending colon, percutaneous endoscopic approach
0DTN4ZZ	Resection of sigmoid colon, percutaneous endoscopic approach

COLOSTOMY

0D1K0Z4	Bypass ascending colon to cutaneous, open approach
0D1L0Z4	Bypass transverse colon to cutaneous, open approach
0D1M0Z4	Bypass descending colon to cutaneous, open approach
0D1N0Z4	Bypass sigmoid colon to cutaneous, open approach
0D1K4Z4	Bypass ascending colon to cutaneous, percutaneous endoscopic approach
0D1L4Z4	Bypass transverse colon to cutaneous, percutaneous endoscopic approach
0D1M4Z4	Bypass descending colon to cutaneous, percutaneous endoscopic approach
0D1N4Z4	Bypass sigmoid colon to cutaneous, percutaneous endoscopic approach

ILEOSTOMY

0D1B0Z4	Bypass ileum to cutaneous, open approach
0D1B4Z4	Bypass ileum to cutaneous, percutaneous endoscopic approach

ERCPC Data Collection Instrument Perioperative Care PRN

Please enter your hospital or health system.

- ○ Barnes-Jewish Hospital
- ○ OhioHealth - Grant Medical Center
- ○ University of Cincinnati Medical Center
- ○ University of Chicago Hospitals
- ○ University of Kentucky Medical Center
- ○ Good Samaritan Medical Center
- ○ Trinity Health Ann Arbor Hospital
- ○ West Virginia University Medicine / Ruby Memorial Hospital
- ○ Beaumont Hospital - Troy
- ○ Saint Francis Hospital / Emory

Patient demographics and parameters

What was the patient's age (in years)?

____
(numbers only)

What was the patient's gender?

○ Female ○ Male

What was the patient's race?

- ○ Indigenous (American Indian/Alaska Native/First Nations/Inuit/Metis)
- ○ Asian
- ○ Native Hawaiian or Other Pacific Islander
- ○ Black or African American
- ○ White
- ○ Hispanic
- ○ More Than One Race
- ○ Unknown / Not Reported

What was the patient's weight on admission to the hospital (in kg)?

____
(numbers only)

What was the patient's pre-operative eGFR on admission from lab data?

____
(numbers only)

How many ethanol-containing beverages does the patient regularly consume on a weekly basis?	◯ none<br>◯ 1-7 drinks<br>◯ 8-14 drinks<br>◯ greater than 14 drinks<br>(Code as NONE for less than 1 drink per week)
At the time of this encounter, did the patient have a documented drug allergy?	◯ Yes ◯ No
To which drug does the patient have an allergy?	☐ non-penicillin/non-cephalosporin<br>☐ penicillin<br>☐ cephalosporin
What was the primary International Classification of Diseases (ICD) 10 code for this admission?	______________________________
What was the second listed International Classification of Diseases (ICD) 10 code for this admission?	______________________________
What was the third listed International Classification of Diseases (ICD) 10 code for this admission?	______________________________

Did the patient have a cancer diagnosis?	◯ Yes ◯ No
What neoadjuvant therapy did the patient receive?	☐ none ☐ radiation ☐ chemotherapy
Which type of surgical technique was used?	◯ Laparoscopic - manual<br>◯ Converted to open - manual<br>◯ Open - manual<br>◯ Laparoscopic - robotic<br>◯ Converted to open - robotic<br>◯ Open - robotic
On what segment(s) of the colon was the procedure performed?	☐ small intestine<br>☐ appendix<br>☐ cecum<br>☐ ascending colon (including right hepatic flexure)<br>☐ transverse colon<br>☐ descending colon (including left splenic flexure)<br>☐ sigmoid colon<br>☐ rectum<br>(multiple entries allowed)
What type of intraoperative anesthesia was used for the procedure?	☐ Epidural<br>☐ General<br>☐ IV lidocaine continuous infusion<br>☐ IV midazolam<br>☐ IV propofol<br>☐ IV short-acting opioid<br>☐ Spinal - opioid<br>☐ Spinal - opioid / LA<br>☐ TAP block - Long acting LA<br>☐ Wound infiltration - liposomal bupivacaine<br>☐ Wound infiltration - non-liposomal bupivacaine / no epinephrine<br>☐ Wound infiltration - non-liposomal bupivacaine / with epinephrine<br>☐ Wound infiltration - bupivacaine / meloxicam<br>(multiple answers allowed)
What was the estimated blood loss (in mLs) during surgery?	________________________________<br>(numbers only)

What intravenous fluids were administered during hospitalization?
(multiple entries allowed)

☐ saline-containing ☐ lactated ringer's ☐ albumin ☐ packed red blood cells

On what day was intravenous fluid stopped?

○ POD #1 ○ POD #2 ○ POD #3 ○ POD #4 ○ POD #5 ○ POD #6 ○ POD #7
○ POD > #7 ○ unknown

What was the American Society of Anesthesiologists (ASA) score? (I-V)

○ I ○ II ○ III ○ IV
○ V

Within the last 6 months, was iron supplementation given to the patient prior to surgery?

○ Yes ○ No

If iron supplementation was given, what kind of iron and/or erythropoetin/biosimilar was prescribed?

☐ oral ferrous sulfate
☐ oral ferrous gluconate
☐ intravenous iron dextran
☐ intravenous ferric derisomaltose
☐ intravenous ferric carboxymaltose
☐ intravenous ferric gluconate
☐ intravenous ferumoxytol
☐ intravenous iron sucrose
☐ erythropoetin or biosimilar
(multiple answers allowed)

Antibiotic Surgical Site Infection (SSI) Prophylaxis

Was a pre-operative antibiotic administered?

○ Yes ○ No

Was more than one IV antibiotic SSI prophylaxis agent administered?

○ Yes ○ No

What, if any, type of oral mechanical bowel preparation was administered?

☐ None ☐ laxatives ☐ antibiotics

Which oral antibiotic(s) were administered prior to surgery?

☐ ciprofloxacin ☐ clindamycin ☐ erythromycin ☐ metronidazole ☐ neomycin

Which IV antibiotic for SSI prophylaxis was administered FIRST?

○ ampicillin/sulbactam
○ cefazolin
○ cefotetan
○ cefoxitin
○ ceftriaxone
○ cefuroxime
○ ciprofloxacin
○ clindamycin
○ ertapenem
○ gentamicin
○ levofloxacin
○ meropenem
○ metronidazole
○ piperacillin/tazobactam
○ tobramycin
○ vancomycin
○ ampicillin

What was the dose of first IV antibiotic administered?

____
(in milligrams - number only)

What was the time difference between the first IV antibiotic administration and surgical incision (in minutes)
○ Unknown ○ 0-15 minutes ○ 16-30 minutes ○ 31-45 minutes ○ 46-60 minutes
○ > 60 minutes

Which IV antibiotic(s) for SSI prophylaxis was administered in combination with the first antibiotic?	☐ ampicillin/sulbactam<br>☐ cefazolin<br>☐ cefotetan<br>☐ cefoxitin<br>☐ ceftriaxone<br>☐ cefuroxime<br>☐ ciprofloxacin<br>☐ clindamycin<br>☐ ertapenem<br>☐ gentamicin<br>☐ levofloxacin<br>☐ meropenem<br>☐ metronidazole<br>☐ piperacillin/tazobactam<br>☐ tobramycin<br>☐ vancomycin<br>☐ ampicillin<br>(please enter second and third antibiotics here)
What was the IV dose of the second and/or third antibiotic administered?	______________________________<br>(in milligrams - numbers only. Enter second and third doses without a space in between.)
Were IV antibiotics administered post-operatively?	○ Yes ○ No
How many post-operative IV antibiotic DOSES (single or in combination) were administered?	○ 0 ○ 1 ○ 2 ○ 3<br>○ 4 ○ >4
Was there any order change in the record that the antibiotic dose was adjusted for the patient's weight?	○ Yes ○ No
If an order change for weight-adjusted antibiotic prophylaxis was in the record, how was the notation generated?	○ manual entry by pharmacist<br>○ automated entry by computer<br>(Use manual entry by pharmacist for pharmacy to dose order changes)
Was an intra-operative re-dose given?	○ Yes ○ No
Which topical skin preparation was applied prior to the incision?	☐ povidone-iodine ☐ chlorhexidine<br>☐ none
Did the patient receive any type of insulin during hospitalization?	○ Yes ○ No

If the patient was administered insulin, what regimen was given?

- ☐ regular insulin by sliding scale (Aspart, Lyspro, or Glulisine)
- ☐ regular insulin by correction method (Aspart, Lyspro, or Glulisine)
- ☐ insulin glargine
- ☐ insulin determir
- ☐ NPH insulin
- ☐ premixed insulin (70/30, 75/25)
- ☐ regular insulin intravenous (bolus or infusion)

(multiple entries allowed)

What level of SSI was diagnosed post-operatively during hospitalization?

○ Incisional ○ Deep ○ Organ space ○ Multiple sites ○ None

Venous Thromboembolism (VTE) Prophylaxis

Was any pre-operative assessment method used to ○ Yes ○ No determine whether the patient was at-risk for post-operative venous thromboembolism?

Calculate the patient's risk for post-operative VTE risk using the embedded website. If your CPOE system calculated the Caprini score, please enter that value into the question below.

Enter the patient's Caprini score (calculated above or computer-generated).

____

Was pre-operative pharmacologic VTE prophylaxis prescribed?

○ Yes ○ No

Which pre-operative VTE prophylaxis agent was administered?

- ○ dalteparin
- ○ enoxaparin
- ○ tinzaparin
- ○ unfractionated heparin

What was the pre-operative VTE prophylaxis dose (in mg)?

____
(numbers only)

What was the pre-operative VTE prophylaxis dose (in units)?

____
(numbers only)

How many pre-operative VTE prophylaxis doses were administered?

○ 0 ○ 1 ○ 2

Was post-operative in hospital pharmacologic VTE prophylaxis prescribed?

○ Yes ○ No

Which post-operative in-hospital VTE prophylaxis agent was administered?	☐ apixaban<br>☐ dabigatran<br>☐ dalteparin<br>☐ edoxaban<br>☐ enoxaparin<br>☐ rivaroxaban<br>☐ tinzaparin<br>☐ unfractionated heparin<br>☐ warfarin<br>☐ fondaparinux
What was the post-operative in-hospital VTE dose? If two or more anticoagulants were administered during the hospitalization, enter both without a space between the doses.	______________________________<br>(numbers only. Enter first and second doses without a space in between.)
What was the frequency of post-operative in-hospital VTE prophylaxis administration?	☐ daily ☐ q12h ☐ q8h
How many post-operative in-hospital VTE prophylaxis doses were administered?	○ 0 ○ 1 ○ 2 ○ 3<br>○ 4 ○ 5 ○ 6 ○ 7<br>○ >7
Was post-discharge (at home) pharmacologic VTE prophylaxis prescribed?	○ Yes ○ No
Which post-discharge (at-home) VTE prophylaxis agent was prescribed?	○ apixaban<br>○ dabigatran<br>○ dalteparin<br>○ edoxaban<br>○ enoxaparin<br>○ rivaroxaban<br>○ tinzaparin<br>○ unfractionated heparin<br>○ warfarin<br>○ fondaparinux
What was the post-discharge VTE prophylaxis dose?	______________________________<br>(numbers only)
What was the prescribed frequency of post-discharge (at-home) VTE prophylaxis administration?	○ daily ○ q12h ○ q8h

For how many days was post-discharge (at-home) VTE prophylaxis prescribed?

○ 1 - 7 days ○ 8 - 14 days ○ 15 - 21 days ○ 22 - 28 days ○ >28 days

What non-pharmacologic VTE prophylaxis used during hospitalization?

☐ none ☐ patient ambulation ☐ compression stockings ☐ sequential compression device (SCD)

Was there any order change in the record that the patient's weight was used to adjust the dose of any VTE prophylaxis agent at any time?	○ Yes ○ No

Was there any order change in the record that the patient's renal function was used to adjust the dose of any VTE prophylaxis agent at any time?

○ Yes ○ No

If there was any order change in the record that the patient's weight or renal function was used to adjust the dose of any VTE prophylaxis agent, how was the notation generated?

○ manual entry by pharmacist
○ automated entry by computer

(Use manual entry by pharmacist for pharmacy to dose order changes)

What was the time difference between the first VTE prophylaxis dose administration and surgical incision (in hours)?

○ Unknown ○ < 6 hours ○ 6-12 hours ○ > 12 hours

Was post-operative VTE present at any time after the procedure?

○ None ○ Brain ○ Peripheral limb ○ Pulmonary ○ Renal

Post-operative Nausea and Vomiting (PONV) Prophylaxis

Was any pre-operative or intra-operative anti-emetic agent(s) for PONV prophylaxis administered?

○ Yes ○ No

Was any post-operative (PACU and ward) anti-emetic agent(s) for PONV prophylaxis administered?

○ Yes ○ No

Which, if any, of the following medication was administered during hospitalization?

☐ None
☐ acetaminophen, intravenous
☐ alvimopan
☐ dexmedetomidine
☐ famotidine
☐ gabapentin
☐ ketamine bolus
☐ ketamine continuous infusion
☐ magnesium sulfate for pain management
☐ naloxegol
☐ neostigmine
☐ pregabalin
☐ prucalopride
☐ propofol
☐ sugammadex

What non-pharmacologic modalities were used to prevent nausea and vomiting?

☐ none ☐ acupuncture
☐ essential oils ☐ wrist band

Which anti-emetic agent(s) were administered prior to induction? (multiple entries if additional agents used)

- ☐ NONE
- ☐ amisulpride
- ☐ aprepitant
- ☐ dexamethasone
- ☐ dolasetron
- ☐ domperidone
- ☐ droperidol
- ☐ granisetron
- ☐ haloperidol
- ☐ metoclopramide
- ☐ ondansetron
- ☐ palonosetron
- ☐ prochlorperazine
- ☐ promethazine
- ☐ scopolamine patch
- ☐ perphenazine
- ☐ hydroxyzine
- ☐ diphenhydramine

How many pre-operative anti-emetic dose(s) (single or in combination) were administered?

○ 0 ○ 1 ○ 2

Which anti-emetic agent(s) were administered prior to extubation? (multiple entries if additional agents used)

- ☐ NONE
- ☐ amisulpride
- ☐ aprepitant
- ☐ dexamethasone
- ☐ dolasetron
- ☐ domperidone
- ☐ droperidol
- ☐ granisetron
- ☐ haloperidol
- ☐ metoclopramide
- ☐ ondansetron
- ☐ palonosetron
- ☐ prochlorperazine
- ☐ promethazine
- ☐ scopolamine patch
- ☐ trimethobenzamide
- ☐ perphenazine
- ☐ hydroxyzine

Excluding aprepitant and scopolamine patch, what was the time difference between IV pre-operative anti-emetic agent administration and the surgical incision? (in minutes)

○ unknown ○ < 60 minutes ○ 60-120 minutes ○ > 120 minutes ○ not applicable

What dose of aprepitant was administered?

____

What route was aprepitant administered?

○ Intravenously ○ Orally

What was the time difference between aprepitant administration and the surgical incision (in minutes)? (in minutes)

○ unknown ○ < 90 minutes ○ 90-180 minutes ○ > 180 minutes

How many hours before surgery was scopolamine patch applied to the skin?

____

Was scopolamine patch left on the skin post-operatively?	◯ Yes ◯ No
Excluding aprepitant and scopolamine patch, how many post-operative anti-emetic dose(s) (single or in combination) were administered?	◯ 0 ◯ 1 ◯ 2 ◯ 3<br>◯ 4 ◯ 5 ◯ 6 ◯ 7<br>◯ >7
What was the scheduled frequency for post-operative anti-emetic administration?	◯ none ◯ around the clock<br>◯ as needed
If as needed (PRN) anti-emetic agents were prescribed, which one was administered for rescue?	☐ amisulpride<br>☐ dimenhydrinate<br>☐ diphenhydramine<br>☐ dolasetron<br>☐ domperidone<br>☐ droperidol<br>☐ granisetron<br>☐ haloperidol<br>☐ metoclopramide IV<br>☐ metoclopramide PO<br>☐ ondansetron IV<br>☐ ondansetron PO<br>☐ palonosetron<br>☐ prochlorperazine IV<br>☐ prochlorperazine PO<br>☐ prochlorperazine PR<br>☐ promethazine IV<br>☐ promethazine PO<br>☐ promethazine PR<br>☐ trimethobenzamide IV<br>☐ trimethobenzamide PO<br>☐ perphenazine IV<br>☐ perphenazine PO<br>☐ hydroxyzine IV/IM<br>☐ hydroxyzine PO<br>☐ dexamethasone<br>(multiple entries allowed)

Multi-modal pain management

Which non-opioid multi-modal pain management agent(s) were prescribed post-operatively?acetaminophen IV	☐ acetaminophen PO<br>☐<br>☐<br>☐ celecoxib PO<br>☐ diclofenac PO<br>☐ diclofenac topical<br>☐ ibuprofen PO<br>☐ ketorolac PO<br>☐ ketorolac IV<br>☐ lidocaine IV<br>☐ lidocaine patch<br>☐ meloxicam PO<br>☐ meloxicam IV<br>☐ naproxen ibuprofen<br>☐ IV gabapentin<br>☐<br>☐

pregabalin
methocarbamol
NONE

Non-opioid multi-modal pain management agent frequency at a standard adult dose

	every 4 hours	every 6 hours	every 8 hours	every 12 hours	every 24 hours	around the clock	as needed
acetaminophen PO	☐	☐	☐	☐	☐	☐	☐
acetaminophen IV	☐	☐	☐	☐	☐	☐	☐
celecoxib PO	☐	☐	☐	☐	☐	☐	☐
diclofenac PO	☐	☐	☐	☐	☐	☐	☐
diclofenac topical	☐	☐	☐	☐	☐	☐	☐
ibuprofen PO	☐	☐	☐	☐	☐	☐	☐
ketorolac PO	☐	☐	☐	☐	☐	☐	☐
ketorolac IV	☐	☐	☐	☐	☐	☐	☐
lidocaine patch	☐	☐	☐	☐	☐	☐	☐
meloxicam PO	☐	☐	☐	☐	☐	☐	☐
meloxicam IV	☐	☐	☐	☐	☐	☐	☐
naproxen	☐	☐	☐	☐	☐	☐	☐
ibuprofen IV	☐	☐	☐	☐	☐	☐	☐
gabapentin 100 mg	☐	☐	☐	☐	☐	☐	☐
gabapentin 200 mg	☐	☐	☐	☐	☐	☐	☐
gabapentin 300 mg	☐	☐	☐	☐	☐	☐	☐
pregabalin 25 mg	☐	☐	☐	☐	☐	☐	☐
pregabalin 75 mg	☐	☐	☐	☐	☐	☐	☐
pregabalin 100 mg	☐	☐	☐	☐	☐	☐	☐
gabapentin 900 mg	☐	☐	☐	☐	☐	☐	☐
methocarbamol	☐	☐	☐	☐	☐	☐	☐

Use the calculator (CLICK ON WATCH VIDEO BELOW) to convert the PARENTERAL (IV/IM/SC) AND/OR ORAL (PO) opioid administered to ORAL (PO) morphine milligram equivalents (MME) and add the amount to the MME in the boxes provided for each of the next 2 MME questions for procedural /PACU and ward total doses. Select the "converting to" for Morphine (oral chronic).

NOTES: TRAMADOL IS CONVERTED TO MME USING THE FOLLOWING CONVERSION: MULTIPLE DOSE TOTAL BY 0.1 AND ENTER WITH OTHER MMEs FROM THE CONVERTER.

For sufentanil and alfentanil conversion to MME, MULTIPLY THE TOTAL DOSE BY 5, AND ENTER THAT VALUE AS FENTANYL (IN MCG).

For remifentantil conversion to MME, MUTLIPLY THE TOTAL DOSE BY 0.5 AND ENTER THAT VALUE AS FENTANYL,

How much intraoperative morphine milligram equivalents (MME) were administered ORALLY & PARENTERALLY during the procedure and in the PACU?

____
(use the MME calculator provided)

How much post-operative morphine milligram equivalents (MME) were administered by ORALLY & PARENTERALLY after PACU discharge and before hospital discharge (i.e. on the ward or unit)?

____
(use the MME calculator provided)

Was any risk stratification scoring system for PONV ○ Yes ○ No used to assess this patient PRE-OPERATIVELY?

Please use the calculator to enter the Apfel scoring system for PONV risk. If your CPOE system calculated the value, please enter that value in the space below.

Enter the patient's Apfel Score.

____
(Score of 0-4 numbers only - no % sign)

Was PONV present at any time after procedure? ○ Yes ○ No

If PONV was present after the procedure, during what time period did it occur?

- ☐ in PACU
- ☐ < 12 hours on ward/unit
- ☐ 12-24 hours on ward/unit
- ☐ >24 hours on ward/unit

(multiple times can be entered)

Use the following Clavien-Dindo Classification of complications scheme to rate the severity of POCs.

Post-operative, in-hospital complications

	None	I-II	IIIa	IIIb	IVa	IVb	V
Infection	○	○	○	○	○	○	○
Fistula/anastomotic leak	○	○	○	○	○	○	○
Bleeding/hematoma	○	○	○	○	○	○	○
Delayed gastric emptying / ileus	○	○	○	○	○	○	○
Nausea and/or vomiting	○	○	○	○	○	○	○
Cardiopulmonary	○	○	○	○	○	○	○
Liver insufficiency	○	○	○	○	○	○	○
Neurologic	○	○	○	○	○	○	○
Pain	○	○	○	○	○	○	○
Allergy	○	○	○	○	○	○	○
Other	○	○	○	○	○	○	○
Renal	○	○	○	○	○	○	○

Use the following Clavien-Dindo Classification of complications to rate the severity of complications experienced at home.

Post-operative, post-discharge complications (frequency and severity)

	None	I-II	IIIa	IIIb	IVa	IVb	v
Infection	○	○	○	○	○	○	○
Fistula/anastomotic leak	○	○	○	○	○	○	○
Bleeding/hematoma	○	○	○	○	○	○	○
Delayed gastric emptying / ileus	○	○	○	○	○	○	○
Nausea and/or vomiting	○	○	○	○	○	○	○
Cardiopulmonary	○	○	○	○	○	○	○
Liver insufficiency	○	○	○	○	○	○	○

Neurologic	○	○	○	○	○	○	○
Pain	○	○	○	○	○	○	○
Allergy Pancreatitis other	○	○	○	○	○	○	○
Renal	○	○	○	○	○	○	○
Lost to follow up - answer not required -	○	○	○	○	○	○	○
select V if lost	○	○	○	○	○	○	○
	○	○	○	○	○	○	○

What was the Length of Hospital Stay (LOSH) related to primary procedure? (in days)

____

(in days)

Was the patient re-admitted to hospital within up to 7 days post-operatively?

○ Yes ○ No

Was the patient re-admitted to hospital between 8 and 30 days post-operatively?

○ Yes ○ No

Supplementary Materials Table S1 – Surgical technique by anatomical location

Surgical technique (n)	Anatomical location (%) – multiple colonic segments included							
	Small intestine	Appendix	Cecum	Ascending colon	Transverse	Descending colon	Sigmoid	Rectum
**Laparoscopic (243)**	26.5	1.2	18.1	36.6	32.5	30.0	53.9	30.5
**Open (137)**	33.8	3.7	16.0	25.6	19.0	30.7	42.3	30.7
**Robotic (96)**	13.5	8.3	9.4	22.9	14.6	31.2	55.2	35.4
**Totals (476)**	25.6	3.4	15.8	30.7	25.0	30.5	50.8	31.5

Supplementary Materials Table S2 – Surgical technique by LOS, 7- and 30-day readmission

Surgical technique (n)	LOS	7-day readmit rate (%)	30-day readmit rate (%)
**Laparoscopic (243)**	5.6	7.8	6.2
**Open (137)**	6.8	2.9	8.0
**Robotic (96)**	4.2	4.2	11.5
**Totals (476)**	5.7	5.7	7.8

Supplementary Materials Table S3 – Anatomical location by LOS, 7- and 30-day re-admission

Anatomical location (n) (multiple segments included)	Secondary outcomes			*p* value		
	LOS	7-day readmit %	30-day readmit %	LOS	7-day readmit	30-day readmit
**Sigmoid (242)**	5.9	6.6	9.9	0.156	0.368	0.076
**Rectum (150)**	6.0	5.3	7.9	0.009*	0.828	0.900
**Ascending colon (146)**	6.1	7.5	7.5	0.528	0.243	0.897
**Descending colon (145)**	6.4	6.8	10.3	0.329	0.445	0.165
**Small intestine (121)**	6.2	8.1	7.3	0.032*	0.162	0.850
**Transverse (119)**	6.8	8.3	7.5	0.019*	0.137	0.921
**Cecum (75)**	5.3	2.6	6.6	0.964	0.685	0.072
**Appendix (16)**	7.7	0.0	5.9	0.284	0.318	0.817

Supplementary Materials Table S4 – Small versus large colon procedures, antibiotics, and SSI rate

Small intestine and other colonic locations		
Antibiotic / combination (n)	SSI incidence (n)	SSI rate (%)
**Piperacillin/tazobactam (2)**	1	50.0
**Ciprofloxacin/metronidazole (3)**	1	33.3
**Cefoxitin (16)**	3	18.8
**Cefoxitin/ampicillin (9)**	1	11.1
**Cefazolin/metronidazole (30)**	3	10.0
**Cefotetan (15), ertapenem (10), clindamycin/gentamicin (3), cefazolin combinations (other) (3), gentamicin/metronidazole (2), cefazolin (1), levofloxacin (1), levofloxacin/metronidazole (1), none (1)**	0	
**Sub-total (97)**	9	9.3
**Small intestine only**		
**Cefazolin/metronidazole (4)**	1	25.0
**Cefoxitin (9), ertapenem (5), cefotetan (4), cefotetan (double dose) (1), none (1)**	0	
**Sub-total (24)**	1	4.2
**Large intestine only**		
**Cefazolin/metronidazole (124)**	3	2.4
**Cefoxitin (51)**	3	5.9
**Ertapenem (65), cefotetan (33), ampicillin/cefoxitin (30), gentamicin/metronidazole (7), cefazolin (6), metronidazole (5), piperacillin/tazobactam (4), ceftriaxone (3), cefazolin combinations (other) (2), clindamycin/gentamicin (2), ampicillin/sulbactam (1), cefotetan/metronidazole (1), cefotetan (double dose) (2), none (19)**	0	
**Sub-total (355)**	6	1.7
**Total (476)**	16	3.4

Supplementary Materials Table S5 – Common postoperative complications by severity – in-hospital (Clavien-Dindo scale)

**Postoperative complication diagnosed during hospitalization in 476 patients**	Grade (seriousness)				
	I-II	IIIa	IIIb	IV	V
**Pain (277; 58.1%)**	274	3			
**Nausea and/or vomiting (228; 47.9%)**	227	1			
**Delayed gastric emptying/ileus (68; 14.6%)**	56	10	2		
**Bleeding/hematoma (35; 7.4%)**	28	4	2	1	
**Infection (32; 6.7%)**	27	3	2		
**Cardiopulmonary (27; 5.7%)**	21	2	1	2	1
**Renal (23; 4.8%)**	22		1		
**Other (16; 3.4%)**	11	1	3		1
**Fistula/anastomotic leak (12; 2.5%)**	6	2	4		
**Neurologic (5; 1.1%)**	4	1			
**Liver insufficiency (1; 0.2%)**	1				
**Totals (714 POCs in 476 patients)**	677 (94.8)	27 (3.8)	15 (2.1)	3 (0.4)	2 (0.3)

Supplementary Materials Table S6 - Common postoperative complications by severity – post-discharge (Clavien-Dindo scale)

**Postoperative complication diagnosed after discharge in 476 patients**	Grade (seriousness)				
	I-II	IIIa	IIIb	IV	V
**Pain (105; 22.0%)**	105				
**Infection (40; 8.4%)**	30	5	5		
**Nausea and/or vomiting (28; 5.9%)**	24	3	1		
**Other (23; 4.8%)**	18	1	3	1	
**Delayed gastric emptying/ileus (18; 3.4%)**	12	5	1		
**Cardiopulmonary (12; 2.5%)**	8	1	1	1	1
**Fistula/anastomotic leak (10; 2.1%)**	5	1	4		
**Bleeding/hematoma (9; 1.9%)**	8	1			
**Renal (4; 0.8%)**	4				
**Neurologic (3; 0.6%)**	3				
**Allergy (1; 0.2%)**	1				
**Totals (253)**	213 (84.9)	26 (10.3)	15 (5.9)	2 (0.8)	1 (0.4)
**Lost to follow-up (7; 1.5%)**					
